# Integrative transcriptomic and metabolomic profiling reveals dynamic differences in pathogen resistance to twig blight in *Myrica rubra*

**DOI:** 10.1186/s12870-025-07543-1

**Published:** 2025-12-05

**Authors:** Yingyao Liu, Yang Song, Peian Zhang, Quan Zhao, Hui Li, Jun Chen, Xiuzhu Guo, Fayong Li, Dongfeng Liu

**Affiliations:** 1 Zhejiang Institute of Subtropical Crops, Wenzhou, China; 2 Yueqing City Bureau of Agriculture and Rural Affairs, Wenzhou, China

**Keywords:** *M. rubra*, *Pestalotiopsis*, Immune response, Transcriptomics, Metabolomics

## Abstract

**Background:**

Twig blight (TB), caused by *Pestalotiopsis*-like species, induces twig dieback in *Myrica rubra*, leading to substantial economic losses in its production across China and posing a significant threat to *M. rubra* cultivation. To date, the immune response of *M. rubra* to *Pestalotiopsis*-like infections remains poorly understood.

**Results:**

In this study, full-length transcriptomics and untargeted metabolomics were employed to investigate the immune responses of resistant “Dingao” (DA) and susceptible “Dongkui” (DK) *M. rubra* cultivars to *Pestalotiopsis* pathogen infection at early (1DPI) and later (5DPI) stage. The main findings revealed that the resistant DA triggered a rapid and robust immune response at 1DPI, with 1,111 differentially expressed genes (DEGs), including 583 upregulated and 528 downregulated. As the immune response progressed to 5DPI, the number of DEGs in Dingao was reduced to 324 DEGs (114 upregulated and 210 downregulated). In contrast, the susceptible Dongkui cultivar exhibited a weak and delayed defense response. At 1DPI only 99 DEGs (26 upregulated and 73 downregulated) were identified in DK leaves; by 5DPI, a substantial shift occurred in Dongkui samples, with 3,414 DEGs (2,141 upregulated and 1,273 downregulated), showing a late but massive defense response. In Dingao samples, WRKY, AP2/ERF transcription factors (TFs) were the most upregulated TFs at the early immune response stage; by 5DPI, NAC and WRKY families became the most prominently expressed TFs. In Dongkui, notable TF upregulation (including WRKY, MYB and AP2/ERF) was only observed at 5DPI and there were not significantly expression changes at the early immune response stage. Further analysis using untargeted metabolomics revealed that Dongkui exhibited 280 upregulated and 12 downregulated differentially accumulated metabolites (DMs) at 1DPI; 503 and 166 DMs were found to be upregulated and regulated at 5DPI. In Dingao plants, 472 DMs were upregulated and 110 downregulated at 1DPI, whereas at 5DPI, 387 DMs were upregulated and 219 downregulated.

**Conclusions:**

Through a combined analysis of full-length transcriptomics and untargeted metabolomics, we revealed distinct molecular and metabolic responses between resistant “Dingao” (DA) and susceptible “Dongkui” (DK) *M. rubra* cultivars under twig blight stress.

**Supplementary Information:**

The online version contains supplementary material available at 10.1186/s12870-025-07543-1.

## Introduction

Chinese bayberry (*Myrica rubra*), also known as yangmei, is a subtropical fruit tree native to eastern Asia, with a cultivation history of over 2,000 years in China [1]. This fruit is celebrated for its sweet-tart flavor and is enjoyed fresh or used in various food products like juices, wines, jams, and traditional medicines. In addition to its culinary appeal, both the leaves and fruits of *M. rubra* are rich in phytochemicals including tannins, flavonoids, triterpenes, and diarylheptanoids. These components exhibit multiple biological activities, with particularly notable anti-inflammatory effects [2]. Chinese bayberry holds significant economic value, with an annual national production of approximately 1.5 million tons as of 2022. Zhejiang Province is a major producer, yielding around 65 thousand tons with an estimated market value of ¥4.6 billion (approximately US$740 million) [3]. Despite its commercial importance and high nutritional content, the yield and quality of Chinese bayberry are increasingly threatened by diseases caused by various pathogens. These diseases can severely impact both the quantity and quality of the fruit, leading to substantial economic losses.

Twig blight (TB), caused by *Pestalotiopsis*-like species, is one of the most severe diseases affecting Chinese bayberry cultivation in China [4]. The disease leads twig dieback and is widespread across all major Chinese bayberry producing areas. *Pestalotiopsis*, a common plant pathogenic fungus, is well-known for causing leaf blight in various plant species, including tea, Chinese bayberry, apple, and guava, resulting in substantial economic losses worldwide [5–7]. Several *Pestalotiposis* species such as *Pestalotiopsis camelliae*, *Pestalotiopsis chamaeropis*, *Pestalotiopsis kenyana*, *Pestalotiopsis rhodomyrtus*, *Pestalotiopsis menhaiensis* and *Pestalotiopsis sichuanensis* are known to cause the gray blight in tea plants [7]. In *M. rubra*, TB is specially caused by *Pestalotiopsis versicolor* and *Pestalotiopsis microspore* [8].

Typically, once mature, five-celled conidia are released from vegetative hyphae, and spread to new plants. These conidia penetrate host cells through natural openings such as stomata and lenticels, or via damaged tissues. The infection first appears as brown spots on leaves and twigs, which shed their leaves and eventually die within 1 to 4 years [9]. This disease spreads rapidly and poses a serious threat to the development of Chinese bayberry cultivation. Despite being one of the most severe plant diseases in this crop, there is limited knowledge about it [10]. As of now, there are no registered pesticides for the specific prevention and control of twig blight in the field [11, 12].

Some *M. rubra* cultivars, such as Dingao, exhibit significant resistance to TB, while others like Dongkui, the most widely cultivated and important cultivar, are highly susceptible (Table S1) [13–15]. To date, the molecular mechanisms underlying resistance and susceptibility between these cultivars remain poorly understood. Therefore, the most urgent priority for combating this disease and protecting Chinese bayberry cultivation and production is extensive breeding research aimed at deepening our understanding of the genetic basis of disease resistance.

Plants have evolved intricate immune systems to resist pathogen attacks and minimize damage. These defenses are primarily divided into two layers: Pattern-Triggered Immunity (PTI) and Effector-Triggered Immunity (ETI). PTI is triggered when pattern recognition receptors (PRRs) on the plant cell membrane recognize pathogen-associated molecular patterns (PAMPs) or microbe-associated molecular patterns (MAMPs) [16, 17]. This response triggers a signaling cascade that strengthens cell walls, produces reactive oxygen species (ROS), and activates defense-related genes, providing broad-spectrum resistance [18]. On the contrary, ETI is a more precise and strong immune response triggered when plant intracellular receptors recognize specific pathogen effectors that attempt to suppress PTI. ETI typically leads to a stronger, more localized defense response, such as the hypersensitive response (HR), resulting in cell death at the site of infection to prevent disease spread [17], thereby offering targeted protection against specific pathogens. Both PTI and ETI activate various well-characterized downstream signaling pathways, such as mitogen-activated protein kinase (MAPK) cascades, the transcription of defense-responsive genes, and hormone signal transduction.

Plants respond to biotic and abiotic stresses through complex regulatory networks, involving transcription factors, signal transduction, plant hormones and secondary metabolites. Transcriptomics is a powerful tool for identifying gene-level regulatory mechanisms, while metabolomics provides insights into key metabolites such as amino acids, hormones, flavonoids, organic acids, and lipids.

Recently a transcriptomics analysis was performed in *M. rubra* leaves to reveal that exogenous brassinolide (BL) significantly lessen the severity of twig blight disease by increasing the contents of chlorophyll, enhancing the activity of antioxidant enzymes, decreasing malondialdehyde (MDA) content and reactive oxygen species (ROS) accumulation and dramatically upregulating the expression of pathogenesis-related (PR) genes in leaves [19].

In order to investigate the resistance mechanisms of *M. rubra* against *P. microspore* induced twig blight, we conducted an integrative analysis of the transcriptomes and metabolomes of two cultivars: the susceptible ‘Dongkui’ (DK) and the resistant ‘Dingao’ (DA). This approach allowed us to identify vital defense signaling and metabolic pathways in the plant’s immune response to twig blight over time.

Our study provides an integrative view of the resistance of Chinese bayberry to twig blight, enhancing our understanding of the genetic and physiological defences of *M. rubra* against *P. microspora*. These findings offer valuable insights for the sustainable cultivation and breeding of Chinese bayberry, providing genetic and physiological countermeasures.

## Material and methods

### Plant and fungal material, growth conditions, and pathogenicity analysis

The Chinese bayberry *(M. rubra*) plants used in this study were grown in open fields within the germplasm resources nursery of the Zhejiang Subtropical Crop Research Institute, Wenzhou, Zhejiang, China. The cultivation area is located at approximately 28°00’ N, 120°63’ E, with an altitude of 100 m. These plants were cultivated using standard agricultural practices, including regular watering, pest control, and fertilization. Inside the nursery, the commercial cultivar “Dongkui” used in this study was purchased from Wenzhou Ouhai Chashan Xinpan Agriculture Co., Ltd. in 2024, and the commercial cultivar “Dingao” was purchased from Yueqing Hongda Fruit Cultivation Specialized Cooperative in 2024.

The pathogenic fungus, *Pestalotiopsis microspora*, were purchased from Biobw (Beijing, China). For cultivation, the fungus was grown on prune agar medium (per L: 40 mL of prune juice, 1 g yeast extract, 5 g lactose, 5 g sucrose, and 20 g agar with pH adjusted to 6.5) at 28 °C for 4–5 days [20].

No field experiments were conducted for the pathogenicity analysis in this study; these were instead performed indoors. Six one-year-old individual plants were randomly selected from both “Dongkui” and “Dingao” cultivars for pathogenicity, transcriptomic, and metabolomic analyses. Following the method described in our previous manuscript, two leaves were cut from each plant and combined into a single group per cultivar. These were then inoculated with agar under laboratory conditions. For inoculation, a 1mm^2^ PA agar containing *P. microspora* was placed onto the surface of plant leaves through the needle-pierced holes. For negative controls, leaves were pierced and treated with plain PA agar (without the pathogen). Inoculated leaves were placed in transparent flasks and covered with plastic wrap to maintain continuous high humidity in a growth chamber (25 °C ± 2 °C). Photographs of the disease phenotype, including both inoculated and systemic leaves, were taken daily following pathogen inoculation. For each genotype, 10 leaves from three individual plants were evaluated per experiment, with three independent replicates performed [8].

### RNA isolation, sequencing, and bioinformatic analyses

RNA extraction, reverse transcription, library preparation, and sequencing were conducted at Shanghai Majorbio Bio-pharm Biotechnology Co., Ltd. (Shanghai, China). RNA-seq libraries were constructed using the Illumina Stranded mRNA Prep Ligation Kit. Poly(A) mRNA was enriched by oligo(dT) beads, fragmented, and reverse transcribed into cDNA with random hexamer primers. After end repair, phosphorylation, and adapter ligation, ~ 300 bp fragments were selected and amplified with15 PCR cycles. Final libraries were quantified with Qubit 4.0 and sequenced on the Illumina NovaSeq X Plus platform (PE150). Raw paired-end reads were trimmed and quality-checked using fastp [21]. Clean reads were aligned to the *M. rubra* genome (http://www.bayberrybase.cn) [15] in orientation mode using HISAT2 [22], and assembled using StringTie [23] using a reference-based approach.

Gene expression levels were quantified as TPM method using RSEM [24]. Differential expression analysis was analysed by the DESeq2 [25] or DEGseq [26], with DEGs defined as |log2FC|≥ 1 and FDR < 0.05 (DESeq2) or FDR < 0.001 (DEGseq). Functional enrichment analysis was conducted with Goatools for GO terms and Python scipy for KEGG pathways. All the alternative splice events were identified by rMATS [27], only isoforms matching the reference or containing new splice junctions were identified. Splicing differences were categorized into exon inclusion/exclusion, alternative 5′/3′, and intron retention.

### Metabolite extraction, LC–MS/MS analysis, and data analysis

Solid Sample: Metabolites were extracted from 50 mg of leaves tissue with 400 μL methanol: water (4:1, v/v) containing 0.02 mg/ml L-2-chlorophenylalanine (internal standard), followed by grinding, sonication, incubation, and centrifugation. The supernatant was collected for LC–MS/MS analysis. After vortexing, sonication, centrifugation, the supernatant was collected for LC–MS/MS analysis. LC–MS/MS analysis: Metabolite profiling was conducted using a Thermo UHPLC-Q Exactive HF-X system equipped with an ACQUITY HSS T3 column (100 mm × 2.1 mm, 1.8 μm; Waters, USA) at Majorbio BioPharm Technology Co., Ltd. MS conditions: data were collected in both positive and negative ESI modes. Full MS resolution was 60,000, and MS/MS resolution was 7,500. Data were collected in DDA mode with a mass range of 70–1050 m*/z*.

Raw LC/MS data were processed using Progenesis QI (Waters Corporation, Milford, USA) to generate a CSV matrix containing sample IDs, metabolite names, and mass spectral intensities. Peaks from internal standards, known false positives and redundant features were removed. Metabolites were identified via HMDB (www.hmdb.ca/), Metlin (https://metlin.scripps.edu/), and the Majorbio Database. Data analysis was performed on the Majorbio Cloud Platform (cloud.majorbio.com) [28]. Features present in ≥ 80% of samples were retained. Missing values below the quantification limit were imputed with minimum value. Intensities were normalized by sum normalization to correct for technical variation. Variables with RSD > 30% in QC samples were excluded. Finally, data were log₁₀ transformed for downstream analysis.

Multivariate statistical analysis was performed by the R package “ropls” (V1.6.2) for PCA and OPLS-DA, with sevenfold cross-validation to evaluate model quality. Metabolites were considered significantly differentially accumulated if they met the criteria of VIP > 1 and *p <* 0.05.

Differentially accumulated metabolites were mapped to pathways using KEGG (www.genome.jp/kegg). Pathway enrichment was performed using Python’s “scipy.stats” package (https://docs.scipy.org/doc/scipy), which identified significantly enriched pathways based on the functional or pathway classification of metabolites.

## Results

### Dingao and Dongkui present difference responses to *P. microspora* infection

**Fig. 1:**
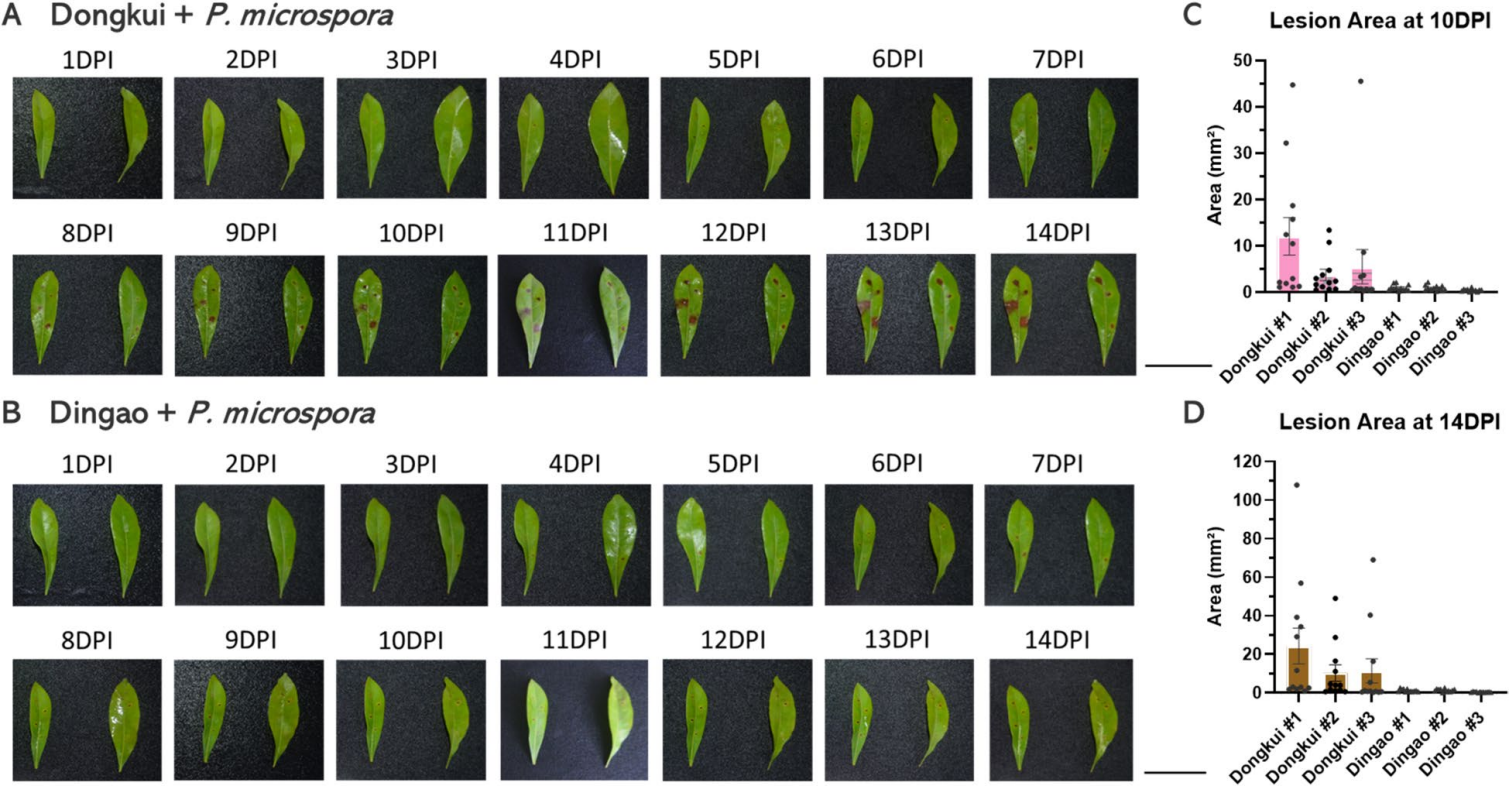
Morphological characteristics and pathogenicity indices of *Myrica rubra* infected with *Pestalotiopsis microspora* at different times. The pathogenic progression of *P. microspora* infection differed significantly between the susceptible cultivar Dongkui (**A**) and the resistant cultivar Dingao (**B**). Scale bar equals to 4 cm. **C** and **D** Quantified disease severity by measuring the lesion area as a pathogenic index after inoculation. We measured the lesions area with three biologic replicates, which showed a significant difference in pathogenic patterns between Dongkui (Susceptible) and Dingao (Resistant). Small dots and triangles represent each lesion on the leaves from three independent replicates

Two Chinese bayberry cultivars were analyzed in this study, including *Pestalotiopsis*-susceptible cultivar Dongkui which shows relatively high susceptible phenotype, and a *Pestalotiopsis* presumed-resistant line Dingao with a strong immune phenotype. First, we conducted plant pathogenicity analysis of the different symptoms induced by *P. microspora*. The young and tender Chinese bayberry leaves were cut and inoculated with agar containing *P. microspora*; we then recorded a series of macroscopic observations to track the pathogenic progress of TB (Fig. 1).

At 1 day post-inoculation (DPI), no symptoms were observed on the wounded leaves of either cultivar. From 5DPI, tiny and brown spots occurred around the *P. microspora* inoculated holes; and the size of these spots increased gradually and progressed to leaf necrosis after 10DPI in Dongkui plants (Fig. 1A). In contrast, few brown lesions were observed in the *P. microspora* inoculated Dingao leaves even after 10DPI (Fig. 1B). The control leaves did not present any symptoms throughout the experiments (Fig. S1 A&B). To further confirm our conclusion, we measured the lesion areas at 10 and 14 DPI. As shown in Fig. 1 C and D and Supplementary Table 2, the quantifying results reveal a clear difference between the two cultivars. Dongkui developed larger and more severe lesions at 10DPI, and the size of thoese lesion contiuned to enlarge by 14DPI after incoulation with *P. microspora*. In contrast, the lesion on Dingao leaves were much smaller and their growth was restrained, indicating Dingao repressed the development of the pathogenic invasion (Fig. 1C, D & Table S2).

### General transcriptome analysis of resistant and susceptible *M. rubra* in response to *P. microspora* infection

To explore the molecular distinctions underlying plant resistance to *P. microspora* infection, eight RNA-seq libraries were first constructed for transcriptomic analysis. These libraries were prepared from the young leaves of the following *M. rubra* cultivars: Dingao (Resistant) and Dongkui (Susceptible). Samples were collected at 1DPI (early infection stage) and 5DPI (later infection stage) based on the macroscopic observations described above (Fig. 1). Additionally, mock-treated leaves at the indicated timepoints served as negative controls.

**Fig. 2:**
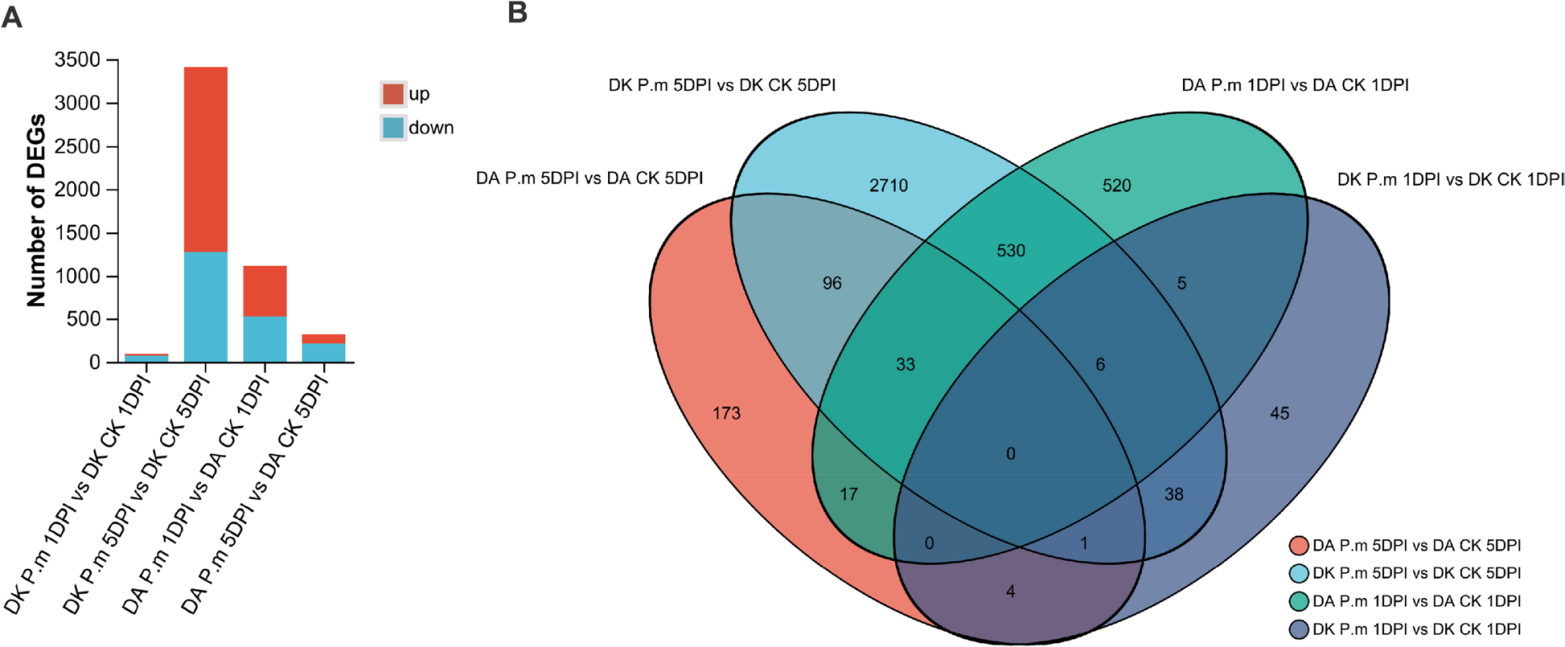
Overview of differentially expressed genes (DEGs) in response to twig blight (TB) at different time points. **A** Number of DEGs under *P. microspora* infection compared with control conditions at two time points; **B** Venn diagram showing common and unique DEGs in *M. rubra* leaves after inoculation with *P. microspora*. At 1DPI, Dongkui showed a distinct gene expression profile, sharing the least number of DEGs with the other samples. In contrast, Dingao at 1 DPI shared 530 DEGs with Dongkui at 5 DPI. The early and late responses within Dingao were also different, as Dingao at 5 DPI shared only 33 DEGs with its 1 DPI sample, but 96 DEGs with the Dongkui 5 DPI samples

Transcriptome sequencing analysis yielded more than seven million clean reads per sample, with the Q20 base percentage exceeding 98.83% (Table S3). A total of 6,818 genes were significantly differentially expressed (DEGs) (Fig. 2A and Table S4). To validate the quality of the RNA-seq results, 50 genes were randomly chosen for expression profiling across treatment groups. A heatmap generated using TBTool [29] presented consistent gene expression patterns, demonstrating a strong correlation within the RNA-seq data (Fig. S2 and Table S5). The fewest DEGs were observed between Dongkui infected with *P. microspora* at 1DPI (DK *P.m* 1DPI) and its control samples (negative control Dongkui leaves harvested at 1DPI), with only 99 DEGs (26 upregulated and 73 downregulated) (Fig. 2A). In contrast, the highest number of DEGs was observed between Dongkui inoculated with *P.m* 5DPI and its control samples, with 3,414 DEGs (2,141 upregulated and 1,273 downregulated). Interestingly, DEGs in Dingao plants showed a rapid response at 1DPI, with 1,111 DEGs (583 upregulated and 528 downregulated). However, by 5DPI, the number of DEGs decreased to 324 DEGs (114 upregulated and 210 downregulated) (Fig. 2A). Our findings indicate a marked difference in the dynamics of gene expression between the susceptible cultivar Dongkui and the resistant cultivar Dingao during *P. microspora* infection.

### Functional annotation and enrichment analysis of DEGs provide insight into contrasting host responses

To analyze the functions of DEGs in response to TB infection, Gene Ontology (GO) classification and enrichment analysis were performed in this study. The GO annotation revealed similar results across samples, with the top five GO annotation terms were consistent, including cellular anatomical entity (GO:0110165), catalytic activity (GO:0003824), binding (GO:0005488), metabolic process (GO:0008152) and cellular process (GO:0009987) (Fig. S3 and Table S6).

**Fig. 3:**
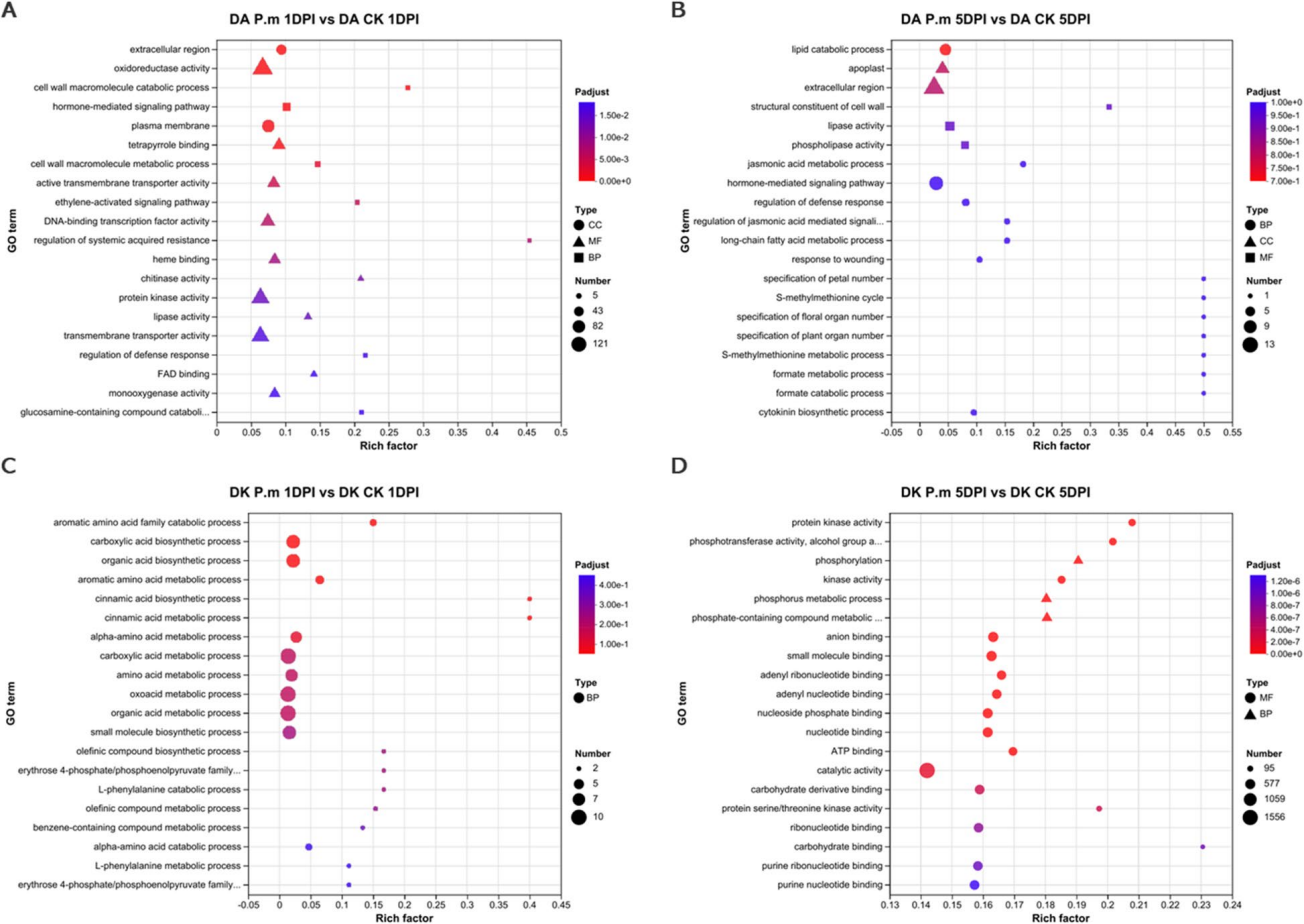
Gene Ontology enrichment of the identified DEGs. GO enrichment analysis identifies different gene expression for 1DPI and 5DPI between Dongkui (DK, Susceptible) and Dingao (DA, Resistant)

GO enrichment analysis revealed significant differences between Dongkui and Dingao samples at different infection stages. In the early infection stage (1DPI) of Dongkui, the DEGs were predominantly associated with biological process (BP), including carboxylic acid metabolic process (GO:0019752), oxoacid metabolic process (GO:0043436) and organic acid metabolic process (GO:0006082). By 5DPI, the number of DEGs in Dongkui increased dramatically, with enrichment primarily in two categories: biological process (BP) and molecular function (MF), catalytic activity (GO:0003824) and various binding-related terms (Fig. 3 and Table S7). In contrast, Dingao samples exhibited a broader range of enriched GO terms across all three categories: BP, MF and CC (cellular component). In Dingao samples at 1DPI, the prominent terms were oxidoreductase activity (GO:0016491), protein kinase activity (GO:0004672) and transmembrane transporter activity (GO:0022857). At 5DPI, the most enriched categories were extracellular region (GO:0005576), hormone-mediated signaling pathway (GO:0009755) and lipid catabolic process (GO:0016042) (Fig. 3 and Table S7).

To better understand the biological functions of these DEGs, we conducted pathway enrichment analysis based on the Kyoto Encyclopedia of Genes and Genomes (KEGG) database. KEGG analysis revealed distinguishable responses between Dongkui and Dingao under *P*. microspora infection. In resistant Dingao samples, the most upregulated pathways during early *P. microspora* infection (1DPI) included the plant MAPK signaling pathway (map04016), plant-pathogen interaction (map04626), plant hormone signal transduction (map04075) and phenylpropanoid biosynthesis (map00940). As the infection progressed to the later stages (5DPI), the number of DEGs in Dingao decreased, but the primary pathways remained plant hormone signal transduction (map04075) and plant MAPK signaling pathway (map04016).

**Fig. 4:**
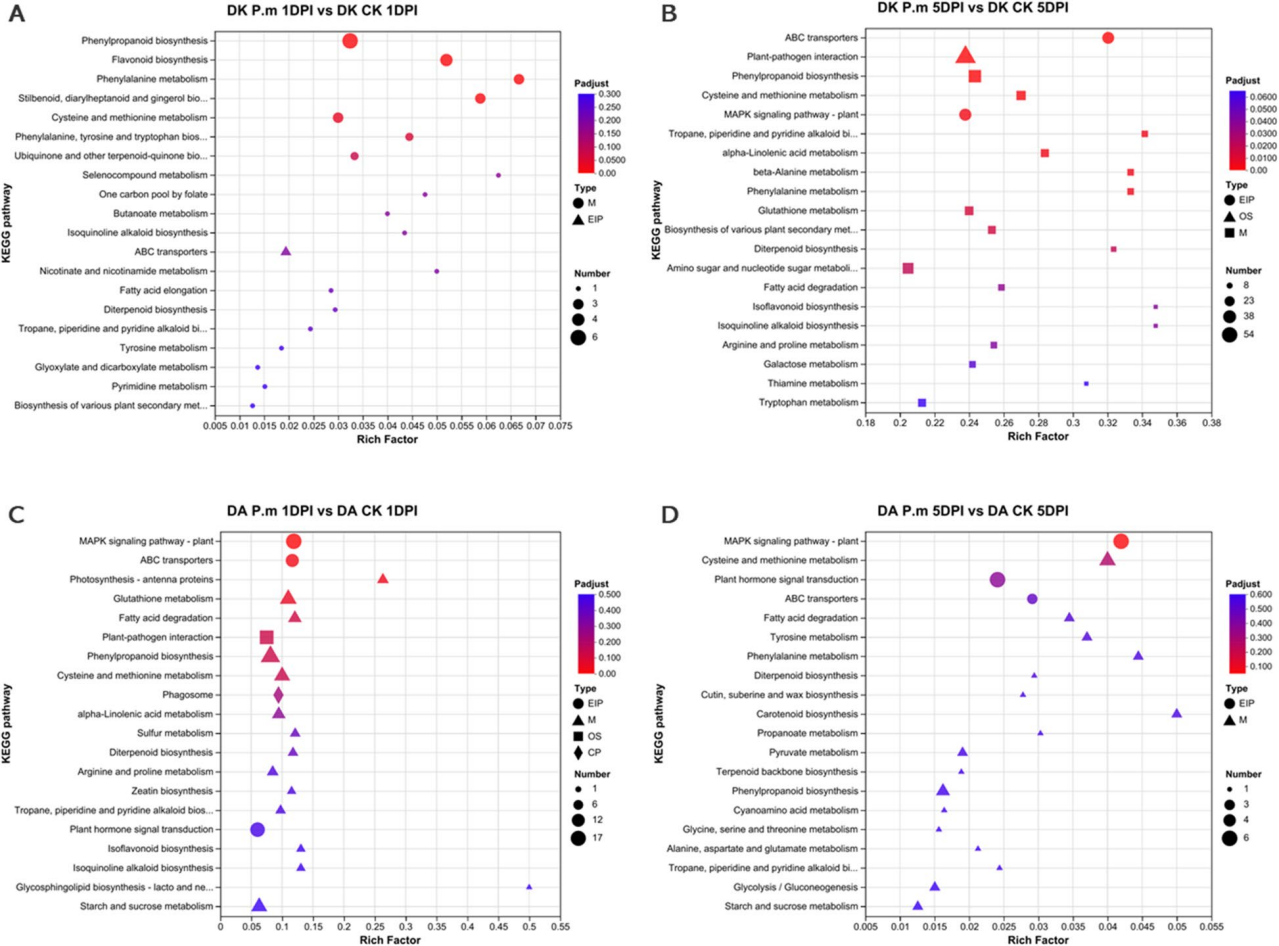
Kyoto Encyclopedia of Gene and Genomes (KEGG) pathways enriched in enriched in DEGs. KEGG pathway profile for 1DPI and 5DPI samples between Dongkui (DK, Susceptible) and Dingao (DA, Resistant)

On the contrary, few DEGs were observed in Dongkui samples at 1DPI, with the main pathways being phenylpropanoid biosynthesis (map00940) and flavonoid biosynthesis (map00941). By 5DPI, as the number of DEGs in Dongkui increased significantly, the most prominently enriched pathways were plant-pathogen interaction (map04626), phenylpropanoid biosynthesis (map00940), plant hormone signal transduction (map04075), and amino sugar and nucleotide sugar metabolism (map00520) (Fig. 4 and Table S8).

Notably, the plant hormone signaling pathway, a vital regulator of plant immune responses, was enriched in Dingao (at both stages) and Dongkui at 5DPI, but not in Dongkui at 1DPI.

### Transcription factors participate in *P. microspora* stress responses in *M. rubra*

**Fig. 5:**
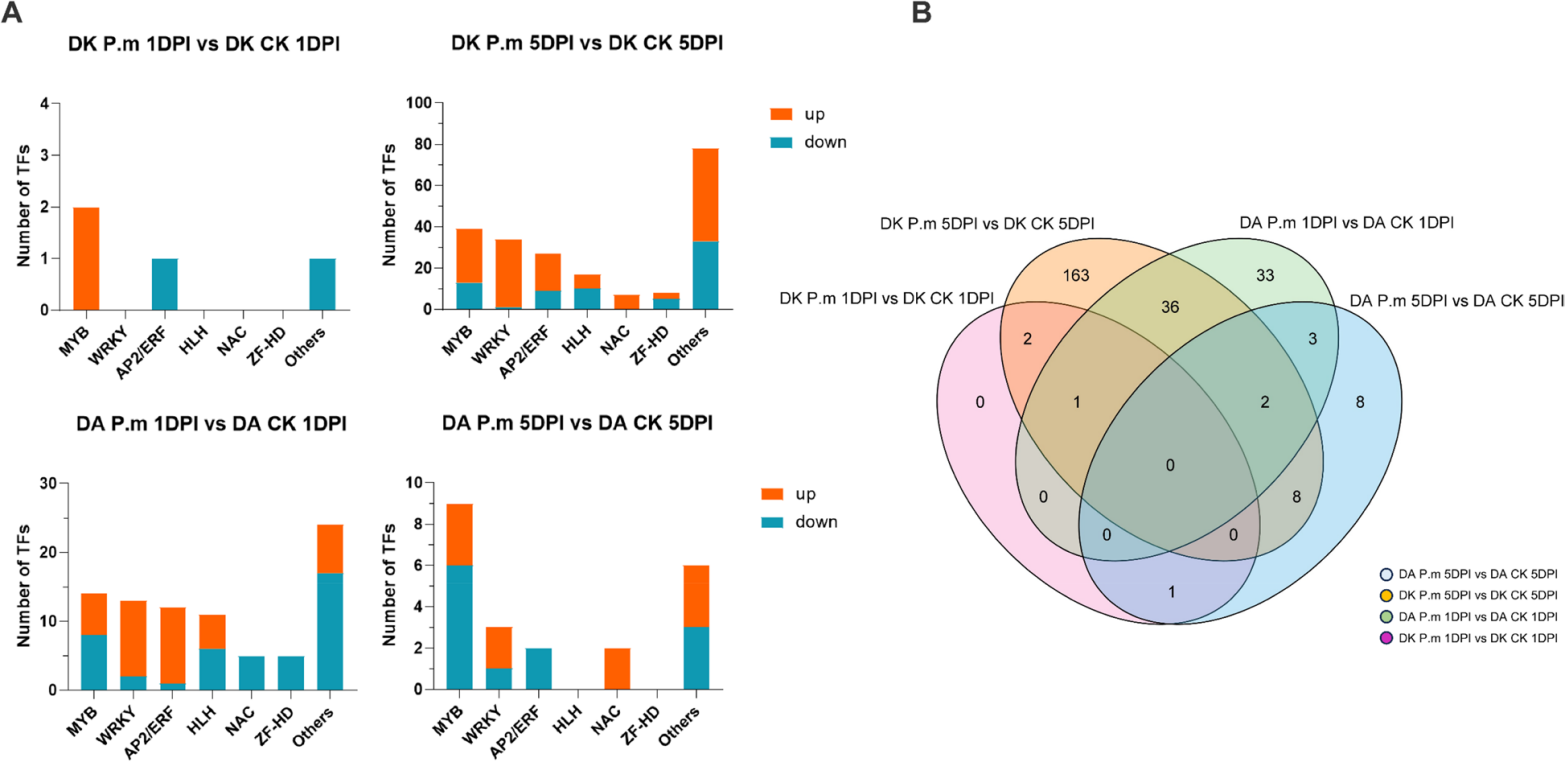
Overview of differentially regulated transcription factors (TFs) in response to twig blight (TB) at different time points. **A** The number of TFs under TB stress compared with control conditions at two time points; **B** Venn diagram diagram showing TFs in Chinese bayberry leaves after inoculation with *P. microspora*

Under *P. microspora* infection, a limited number of shared differentially expressed transcription factors (TFs) were identified between Dongkui (Susceptible) and Dingao (Resistant) (Fig. 5B). In Dingao, the resistant cultivar, the most significantly changed TF families at 1DPI were MYB, WRKY, AP2/ERF, and HLH; most of WRKY and AP2/ERF TFs were upregulated. By 5DPI, most MYB and AP2/ERF TFs were downregulated; while the expression levels of NAC TFs were upregulated markedly. However, Dingkui exhibited only three TFs at 1DPI: one from the MYB family was upregulated while the other from the AP2/ERF family was downregulated. By 5DPI, the number of expressed TFs increased significantly, with the most upregulated families being MYB, WRKY, AP2/ERF, HLH, and NAC (Fig. 5A).

While WRKY and AP2/ERF family TFs in Dingao samples at 1DPI, as well as WRKY family TFs in Dingkui samples at 5DPI, were predominantly upregulated, the expression patterns of other TF families were more complex, some TFs in the same family being upregulated and others downregulated (Fig. 5 and Table S9). Notably, NAC family TFs were upregulated in the later stage (5DPI) in both Dongkui and Dingao samples, but were downregulated at 1DPI in the resistant Dingao samples (Fig. 5A). This suggests that NAC TFs play a distinct and significant role in the *P. microspora* stress response in *M. rubra*. Additionally, the WRKY TFs upregulated in Dingao 1DPI leaves were also upregulated in Dongkui 5DPI leaves. This coordinated upregulation across different time points in different varieties suggests a shared defense mechanism and underscores the critical role these WRKY TFs play in the peak immune response of *M. rubra*.

### Overview of untargeted metabolomics

**Fig. 6:**
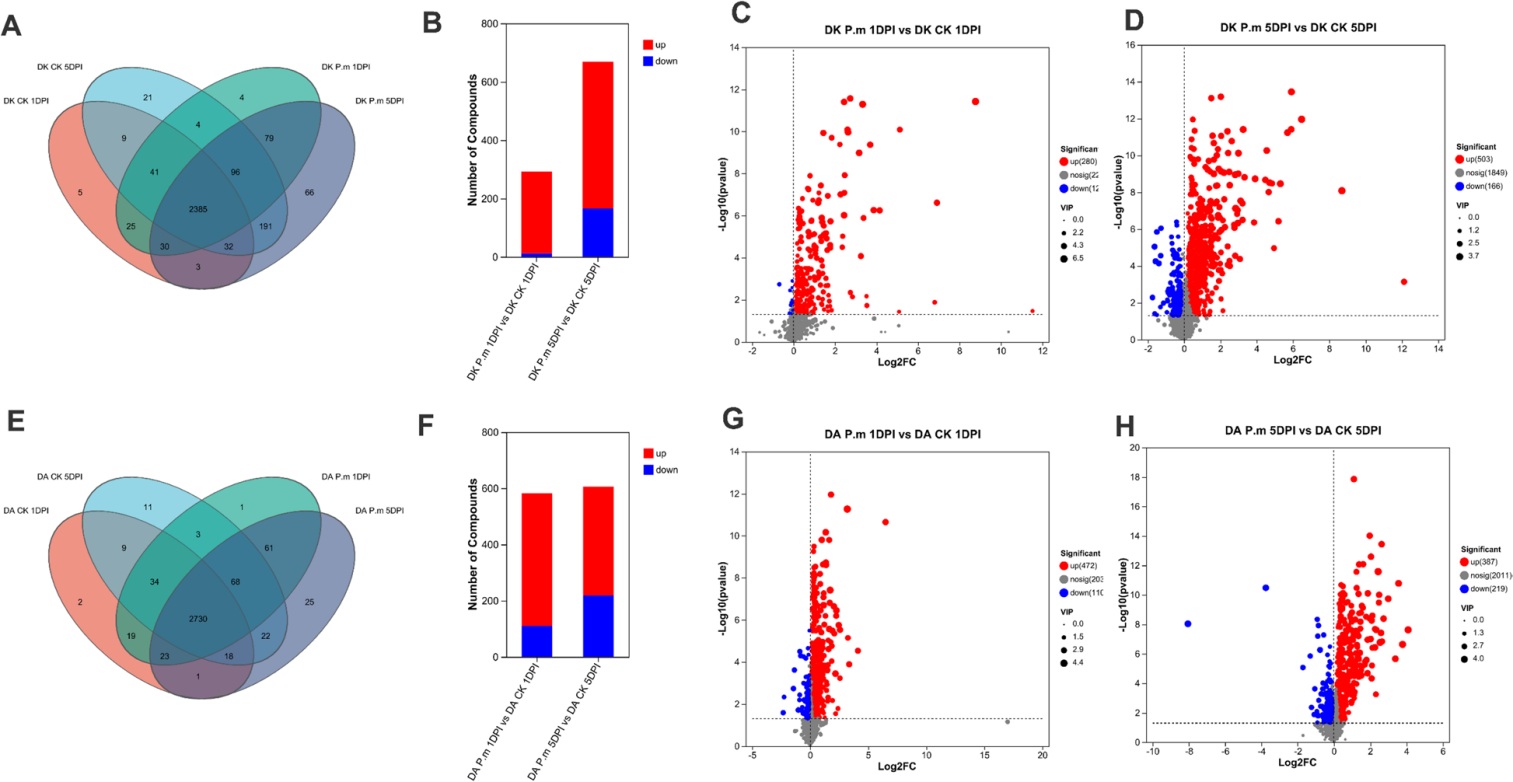
Overview of metabolomic analysis. Differential metabolite abundances identified in comparisons between different treatments and stages (**A**, **B**, **E**, and **F**). Volcano plots of differential metabolites for *P. microspora*-treated groups vs. CK groups (**C**, **D**, **G**, and **H**). The x-axis shows log₂ fold change in metabolite abundance between groups, and the y-axis shows –log₁₀ P-value indicating significance. Each dot represents a metabolite. Higher values indicate greater significance in differential expression. Each dot indicates an individual metabolite

To further investigate the metabolite changes under the *P. microspora* attack in *M. rubra*, we performed untargeted metabolomic analyses for the control and infested Dongkui and Dingao leaves. A total of 2,512 (1,518 positive and 994 negative) and 2,611 (1,647 positive and 964 negative) annotated metabolites were detected from Dongkui and Dingao samples in comparisons between *P. microspora*-treated and control sample, respectively (Fig. 6). Principal component analysis (PCA) revealed that PC1, PC2, and PC3 contained 38.70%, 18.60%, and 10.80% of metabolites in the Dongkui samples, respectively; and for 42.00%, 16.70% and 9.97% of the variance (Fig. S4 A&D), samples were clearly separated in the PCA models. Partial Least Squares Discriminant Analysis (PLS-DA) revealed clear separation of metabolites among all groups, except for the two Dingao mock treatments (Fig. S4 B&E), suggesting that *P. microspora* treatment significantly perturbed *M. rubra* metabolite profiles. The PLS-DA model validation results showed that R^2^ values exceeded Q^2^ in both models, with Q^2^ regression line intercepts on the Y-axis were − 0.9410 and − 0.8925, respectively (Fig. S4 C&F). These results indicate good model fit, high predictive ability, and suitability for subsequent data analysis.

### Differential metabolites responses to *P. microspora* stress

To identify the core metabolites involved in immune responses to *P. microspora* stress, we compared metabolites between the *P. microspora*-inoculated and control groups, and this analysis enabled the identification of specific differentially abundant metabolites (DMs).

At 1DPI, the Dongkui *P. microspora*-inoculated group exhibited 280 upregulated and 12 downregulated DMs. At 5 DPI, Dongkui infected with *P. microspora* showed 503 DEGs that were upregulated and 166 that were downregulated compared to control samples. In Dingao plants, 472 DMs were upregulated and 110 downregulated at 1DPI, whereas by 5DPI, the number of DMs in Dingao samples shifted to 387 DMs were upregulated and 219 downregulated (Fig. 6 and Fig. S5).

A comparison of DMs between Dongkui and Dingao samples showed that Dingao, the resistant cultivar, mounted a faster immune response to *P. microspora* attack. Interestingly, the dynamic DM patterns of DMs closely mirrored the gene expression patterns in both the susceptible cultivar Dongkui and the resistant cultivar Dingao during *P. microspora* infection.

### Analysis of the association between KEGG and metabolic data in responses to the *P. microspora* stress

To investigate the mechanisms underlying resistance to *P. microspora* infection, we analyzed the expression patterns of immune-related genes alongside their associated metabolites across key metabolic pathways.

**Fig. 7:**
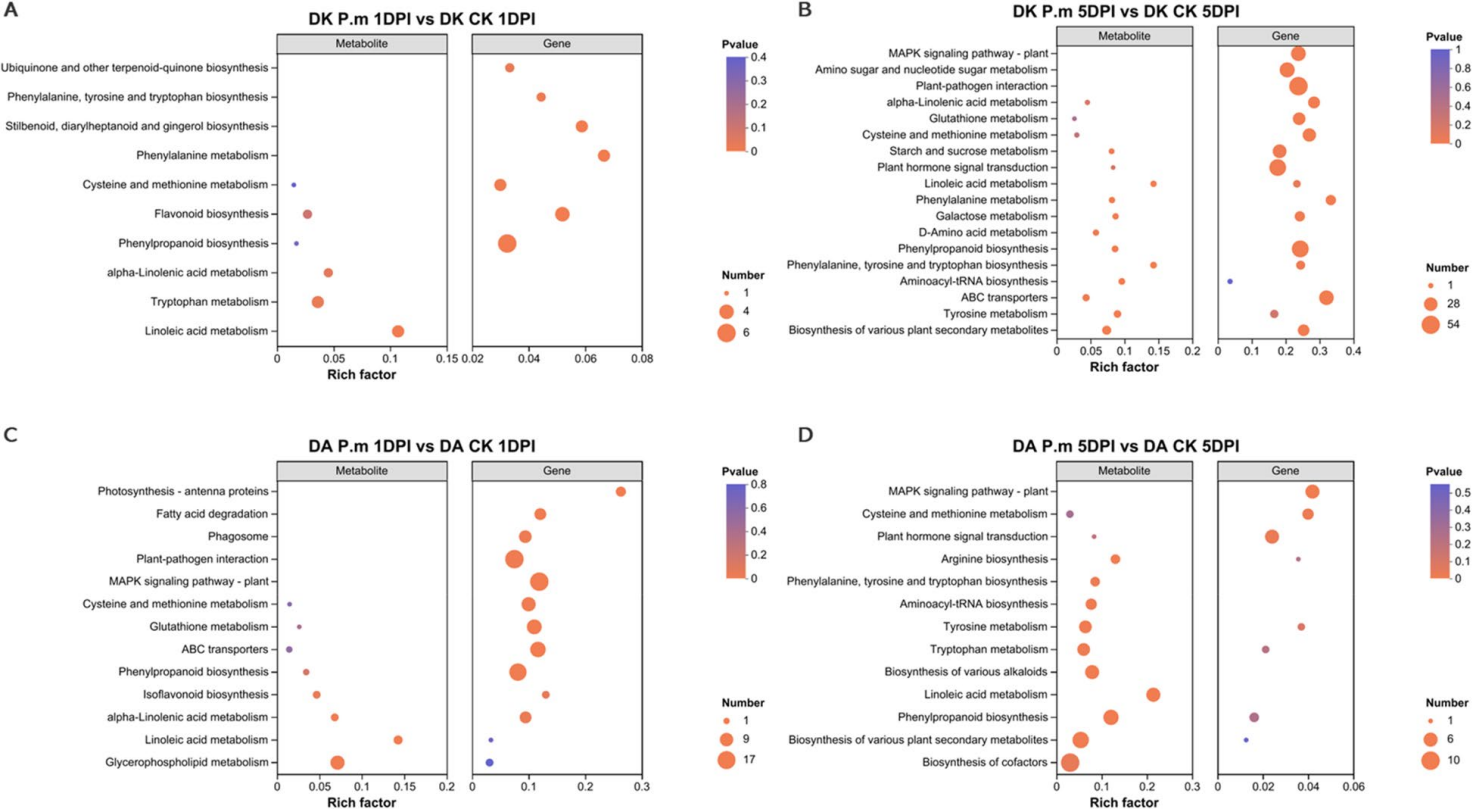
KEGG enrichment of the differential metabolites. **A** Metabolites identified in Dongkui 1DPI samples. **B** Metabolites identified in Dongkui 5DPI samples. Dot size reflects to the number of DMs in each pathway, while color indicates the enrichment significance. Ratio refers to the number of metabolites in each pathway relative to the total number of enriched metabolites

Significant DMs in Dongkui and Dingao were categorized according to the pathway classifications in the KEGG database (Fig. 7). In the resistant Dingao samples, by 1DPI, the top metabolic pathways were: linoleic acid metabolism, glycerophospholipid metabolism, and porphyrin metabolism. By 5DPI, these shifted to biosynthesis of cofactors, biosynthesis of various plant secondary metabolites and phenylpropanoid biosynthesis. In the susceptible Dongkui samples, the top three enriched pathways were: linoleic acid metabolism, tryptophan metabolism and biosynthesis of various plant secondary metabolites at 1DPI. By 5DPI, the most enriched metabolic categories had shifted to biosynthesis of various plant secondary metabolites, tyrosine metabolism and ABC transporters.

These results suggest that resistance to *P. microspora* in Dingao may be attributed to the early activation of lipid and porphyrin metabolism, followed by the induction of secondary metabolite and phenylpropanoid biosynthesisn processes that play critical roles in the plant immune response. In contrast, the delayed or altered metabolic responses in the susceptible Dongkui, including the enrichment of tryptophan and tyrosine metabolism and ABC transporters, may reflect a less effective or misregulated defense strategy.

## Discussion

### Dongkui and Dingao have distinct DEGs patterns in comparative transcriptomic analysis under *P. microspora* attack

Over thousands of years of evolution, plants have developed complex and sophisticated immune responses to counter attacks from pathogenic microorganisms. In our study, we confirmed the distinct disease phenotypes between a susceptible and a resistant *M. rubra* variety when challenged by *P. microspora*. Transcriptomic analysis revealed significant differences in immune responses between the resistant variety (Dingao) and the susceptible variety (Dongkui).

The most striking difference was the number of DEGs at 1DPI (Fig. 2). Dingao exhibited a rapid and robust defense response, triggering thousands of DEGs at 1DPI, whereas Dongkui showed few DEGs at this early stage. Although Dongkui regulated more DEGs at a later stage (5DPI), this delayed response was unable to effectively contain pathogen progression. Based on our observations and analysis, we propose that the early immune response (1DPI) in *M. rubra* represents a critical window for repressing the development of Twig Blight in this species.

### Signaling pathways and transcription factors involved in immune responses to *P. microspora* stress

During pathogen invasion, plant immune responses including PTI and ETI trigger multiple signaling pathways for the resistance responses, including mitogen-activated protein kinases (MAPKs), defense hormones signalling pathways and calcium-dependent protein kinases (CDPKs), and so on [30, 31]. In our study, hundreds to thousands of DEGs in *M. rubra* were identified upon inoculation with *P. microspora*. Our research revealed that in the resistant Dingao samples, the predominant pathways throughout TB infection were plant MAPK signalling pathway and Plant hormone signal transduction, pathways that were absent in Dongkui at the early infection stage.

We further analyzed the transcription factors involved in the pathogenic process in *M. rubra*, as they play essential roles in regulating gene expression during plant immune responses.

Our investigation discovered that WRKY and AP2/ERF families were the most upregulated TFs in Dingao during early-stage immune responses. Overtime, the most upregulated TFs belonged to NAC and WRKY families at 5DPI. In Dongkui, by 5DPI, the most upregulated TFs families were WRKY, MYB, and AP2/ERF, which were not detected at the early stage (1DPI). In addition, we noticed that the expression levels of NAC family TFs were increased in both cultivars at 5DPI.

In plants, WRKY TFs regulate a wide range of biological functions, including receptor-mediated pathogen-triggered immunity [32], chromatin modulation for specific gene interactions [32], and signal transduction through complex gene networks [32, 33]. AP2/ERF transcription factors initiate abiotic stress-responsive through both abscisic acid (ABA)-dependent and ABA-independent pathways, responding to hormones such as abscisic acid, auxin, ethylene, and gibberellin [34]. MYB TFs regulate the expression of resistance genes, hormone defense signaling, lignin/flavonoid/cuticular wax biosynthesis, and the hypersensitivity response [35]; NAC TFs play extensive roles in stress responses and disease resistance, acting through diverse and complex plant hormone signaling pathways involved in defense responses [36]. Combining our KEGG and TFs analyses, our results highlight the importance of plant hormones in *M. rubra* in response to *P. microspora.*

### Plant hormones and secondary metabolism are altered in response to *P. microspora* attack

Plant hormones play a crucial role in defense against pathogens, becoming activated upon the recognition of microbial or insect-derived molecules to initiate downstream defense signaling pathways through both PTI and ETI [37, 38]. Among these hormones, jasmonic acid (JA) and salicylic acid (SA) are the primary defense phytohormones in plant defense responses against pathogens [39, 40].

The KEGG pathway analysis revealed that Plant hormone signaling transduction was a dominant pathway in both Dingao 1DPI and 5DPI samples, while it was absent in the Dongkui samples (Fig. 4). Furthermore, Dingao samples showed α-linolenic acid metabolism as a dominant metabolic pathway at 1DPI (Fig. 4 C). α-linolenic acid is a precursor in the oxylipin pathway, which leads to the production of jasmonic acid (JA). This suggests a potentially important role JA play in countering the attack from *P. microspora*.

Further KEGG enrichment analysis of the differential metabolites showed that α-linolenic acid metabolism and linoleic acid (a precursor of α-linolenic acid) metabolism were upregulated at 1DPI in the *P. microspore* treated samples (Fig. 7 A&C). At 5DPI, both pathways reamined activated in Dongkui samples, but only linoleic acid metabolism upregulated in Dingao’s samples (Fig. 7 B&D).

**Fig. 8:**
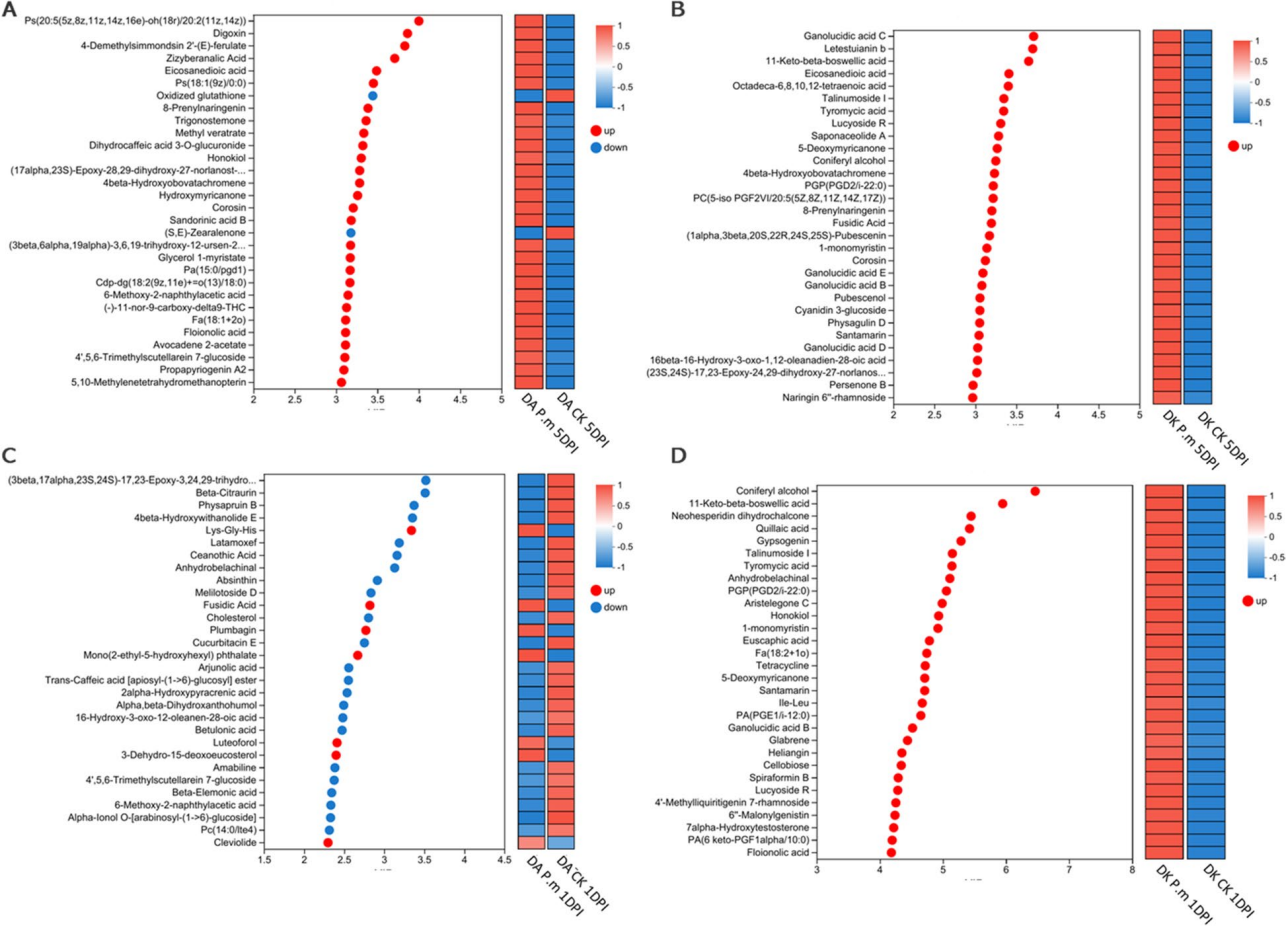
Differentially abundant metabolites related to the plant defense responses. VIP bar chart of differentially abundant metabolites. The default threshold for VIP values is ≥ 1. The larger the VIP value, the greater the contribution of the metabolite to the differences between the two groups. Bar color indicates the significance of this difference: the smaller the *P*-value, the darker the color

The list of these metabolites, presented in Supplementary Table S11, shows that although the director precursor of JA, α-linolenic acid was significant upregulated in *M. rubra* samples following the *P. microspore* attack, only the Dingao cultivar synthesized a remarkable amount of JA at 1 DPI (Table S11). Previous studies have found that JA can suppress the pathogenicity of *P. microspora* in *M. rubra*, though the underlying molecular mechanisms remain unclear [41]. Our untargeted metabolomics results revealed trace amounts of jasmonic acid (JA) under normal conditions, and its biosynthesis was further enhanced following *P. microspora* attack at 1 DPI in the pathogen-resistant cultivar (Table S10), highlighting the importance of JA singaling in the resitent to this pathogenity progress (Fig. 8).

Transcriptomic data revealed that the *MYC2* (GENE_006112), a master regulator of responses, was upregulated in Dingao 1DPI with *P. microspore* treated leaves and downregulated at 5DPI stages. Similarly, two *JAZ* genes (GENE_013815 and GENE_025552) were upregulated at 1DPI in Dingao leaves. In contrast, Dongkui leaves at 5DPI following the *P. microspora* treatment, showed upregulation of two different *JAZ* genes (GENE_013815 and GENE_013627), while a third *JAZ* gene (GENE_020363) was downregulated (Table S4).

The observed pattern of *MYC2* and *JAZ* genes expression suggests a complex and time-dependent regulation of the JA signaling pathway in response to *P. microspora* infection. Since JAZ proteins typically bind to and inhibit TFs like MYC2, the changes in their expression levels are a strong indicator of an active defense response. The temporal differences in JA accumulation align with the defense patterns in our transcriptomic data, indicating that *M. rubra* synthesizes and accumulate JA to counter *P. microspora* infection. This finding highlights the crucial role of the JA pathway in the plant's defense against *P. microspora*.

Taken together, we propose that the early conversion of linoleic acid to jasmonates in Dingao represents a critical defense stage, enabling the plant to promptly resist the invasion by the pathogenic fungus *P. microspora*. Taken together, our findings highlight the significant role that jasmonates play in the defense response of *M. rubra* against twig blight disease.

## Conclusions

To summarize, by conducting comparative transcriptional profiling and metabolic analysis of two *M. rubra* cultivars: the resistant Dingao and susceptible Dongkui following the inoculation with *P. microspora*, we identified 8,399 and 2,313 shared DEGs at 1DPI and 5DPI, respectively. Additionally, we observed 2,512 and 2,611 DMs at 1DPI and 5DPI, respectively, between Dingao and Dongkui under *P. microspora* stress.

**Fig. 9:**
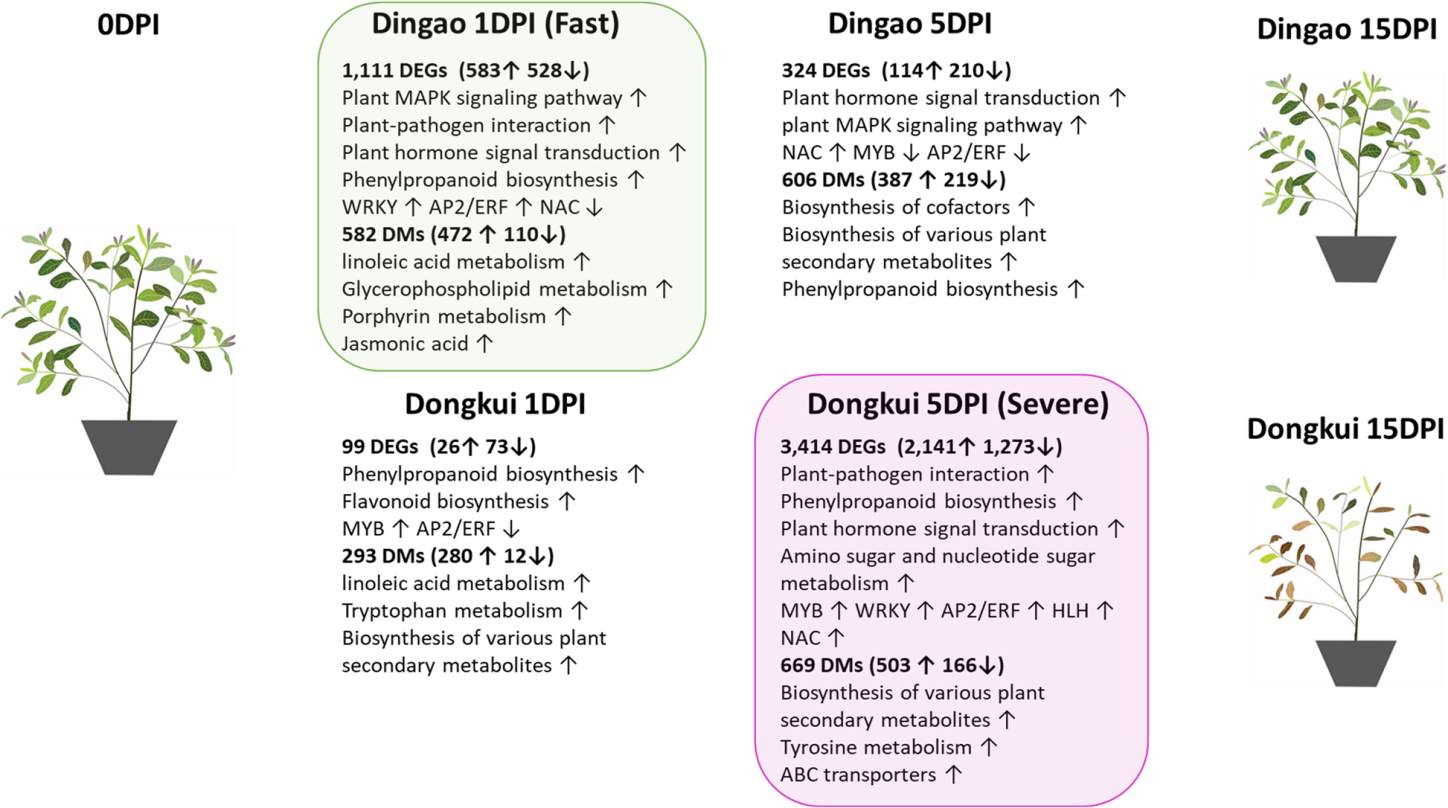
The Different plant defense responses in Dingao and Dongkui resulted in contrasting levels of resistance. In summary, the resistant cultivar Dingao triggers a fast and effective defense response to counter *P. microspora* infection. In contrast, the susceptible cultivar Dongkui presents a delayed but severe immune response that fails to halt the infection’s progress

The most notable difference between Dingao and Dongkui was that the resistant Dingao cultivar mounted a rapid and robust immune response to *P. microspora*’s invasion at 1DPI. Furthermore, Dingao exhibited jasmonate biosynthesis at this early stage (Table S10), a phytohormone previously reported to suppress TB in *M. rubra* (Fig. 9).

Our findings enhance the understanding of molecular defense mechanisms in *M. rubra* and represent important progress toward developing disease-resistant cultivars through molecular breeding.

## Supplementary Information

Supplementary Material 1.

Supplementary Material 2: Fig. S1. Morphological characteristics and pathogenicity indices of *Myrica rubra *infected with plain agar (negative control) at different times. Fig. S2 Heatmap of DEGs expression levels. Fig. S3 Gene Ontology classification of identified DEGs. Fig. S4 Quality analysis of metabolomics data.

## Data Availability

Sequence data that support the findings of this study have been deposited with the primary accession code PRJCA042809 at the China National Center for Bioinformation/Beijing Institute of Genomics, Chinese Academy of Sciences, and are publicly available at https://ngdc.cncb.ac.cn/genbase. All the data are public available and can be accessed at any time.

## Abbreviations

BL: Brassinolide
BP: Biological Process
CDPK: Calcium-dependent Protein Kinase
DA: Dingao
DEG: Differentially Expressed Gene
DK: Dongkui
DM: Differentially accumulated metabolite
DPI: Day post-inoculation
ETI: Effector-Triggered Immunity
GO: Gene Ontology
HR: Hypersensitive Response
JA: Jasmonic Acid
KEGG: Kyoto Encyclopanoid of Genes and Geneomes
MAMP: Microbe-associated Molecular Pattern
MAPK: Mitogen-activated Protein Kinase
MDA: Malondialdehyde
PAMP: Pathogen-associated Molecular Pattern
PCA: Principal Component Analysis
PLS-DA: Partial least squares discriminant analysis
PTI: Pattern-triggered Immunity
ROS: Reactive Oxygen Species
SA: Salicylic Acid
TB: TWIG Blight
TF: Transcription Factor
