# Supplementary material for: Integrative transcriptomic and metabolomic profiling reveals dynamic differences in pathogen resistance to twig blight in Myrica rubra
Source: BMC Plant Biol. 2025 Dec 5;25:1681. doi: 10.1186/s12870-025-07543-1 (PMC12681098; doi:10.1186/s12870-025-07543-1)
Supplement: Supplementary file 2 — Supplementary Material 2: Fig. S1. Morphological characteristics and pathogenicity indices of Myrica rubra infected with plain agar (negative control) at different times. Fig. S2 Heatmap of DEGs expression levels. Fig. S3 Gene Ontology classification of identified DEGs. Fig. S4 Quality analysis of metabolomics data. [file 12870_2025_7543_MOESM2_ESM.docx]

Fig. S1 Morphological characteristics and pathogenicity indices of *Myrica rubra* infected with plain agar (negative control) at different times.


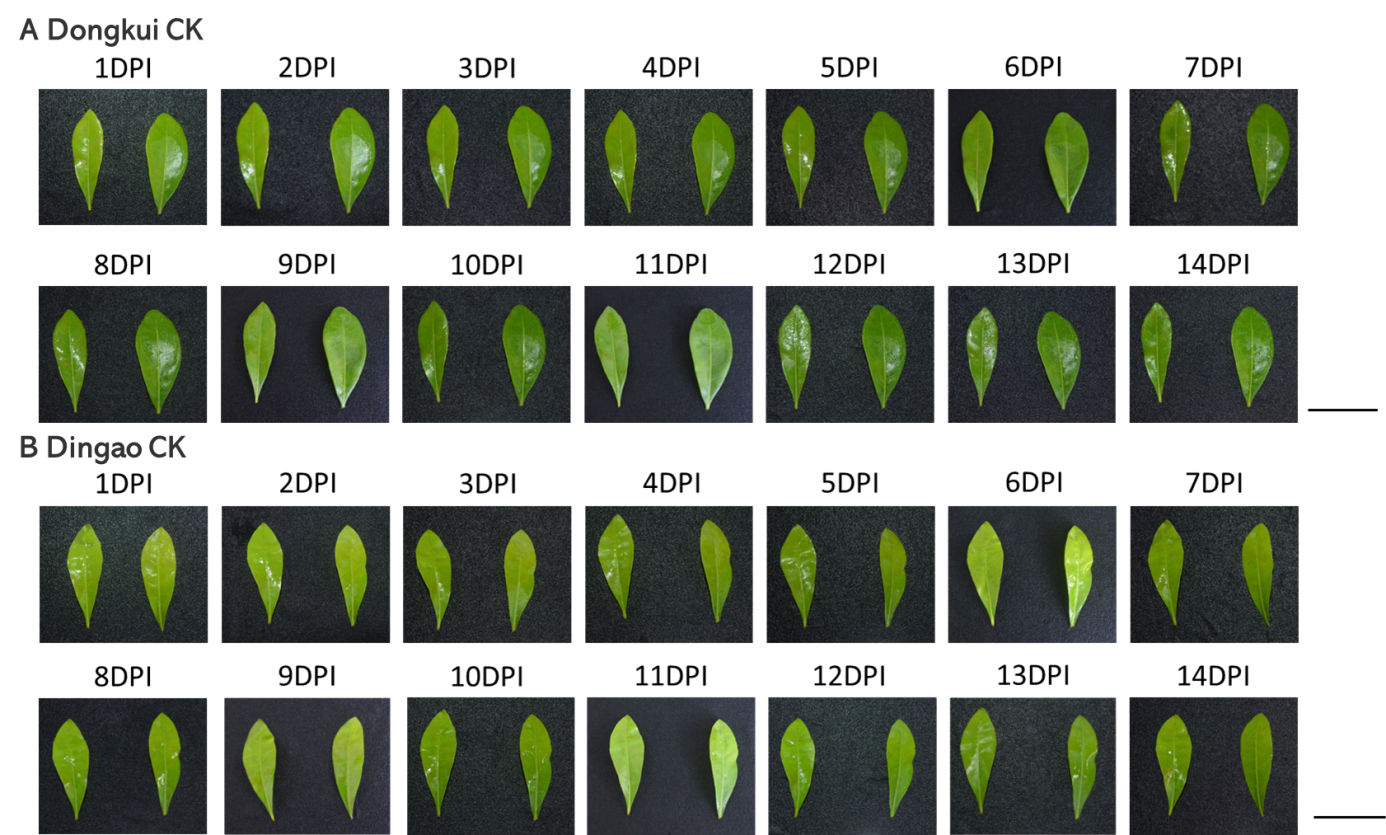


The progression of plain agar inoculation in the susceptible cultivar Dongkui (A) and the resistant cultivar Dingao (B), serving as a negative control to assess baseline responses in the absence of *P. microspora* infection. Scale bar equals to 4cm.

Fig. S2 Heatmap of DEGs expression levels.


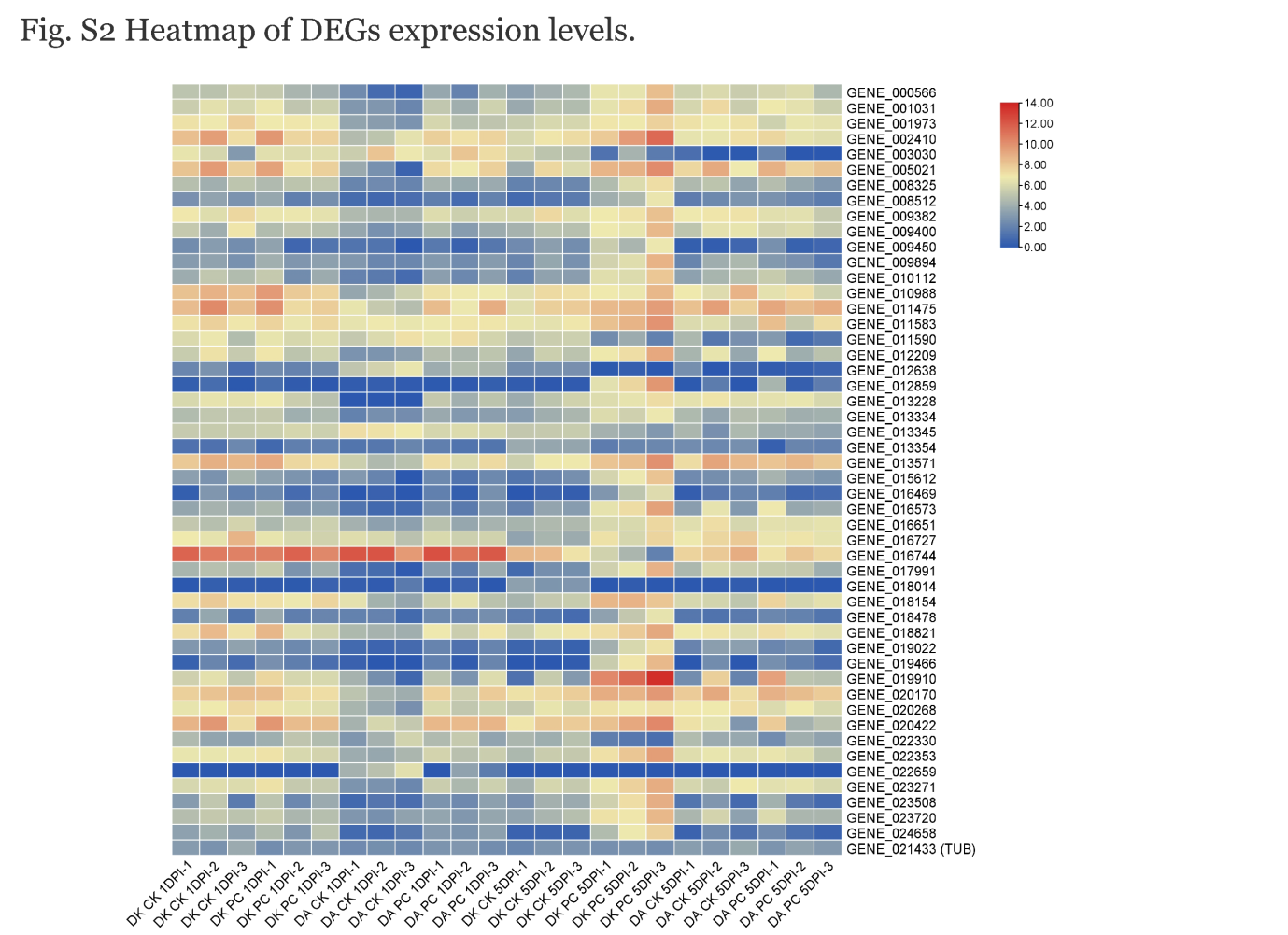


Heatmap showing the expression levels of 50 randomly selected DEGs based on the largest log fold change (logFC) values. Each tile indicates the expression level of a gene in a specific sample. Deeper red indicates higher expression, while deeper blue indicates lower expression. FC: fold change.

Fig. S3 Gene Ontology classification of identified DEGs.


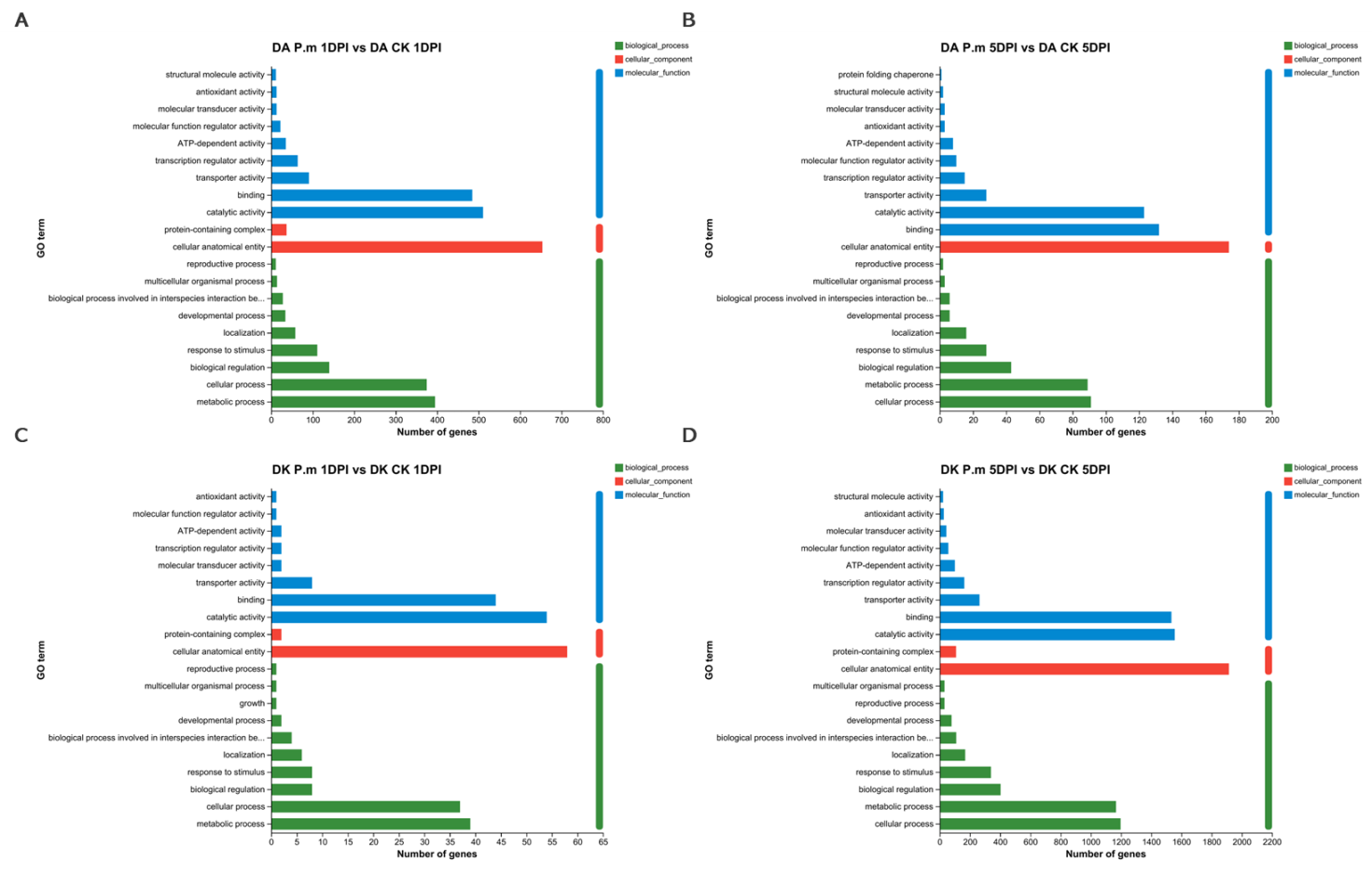


GO classification distribution for 1DPI and 5DPI samples between Dingao (DA, resistant) and Dongkui (DK, susceptible).

Fig. S4 Quality analysis of metabolomics data.


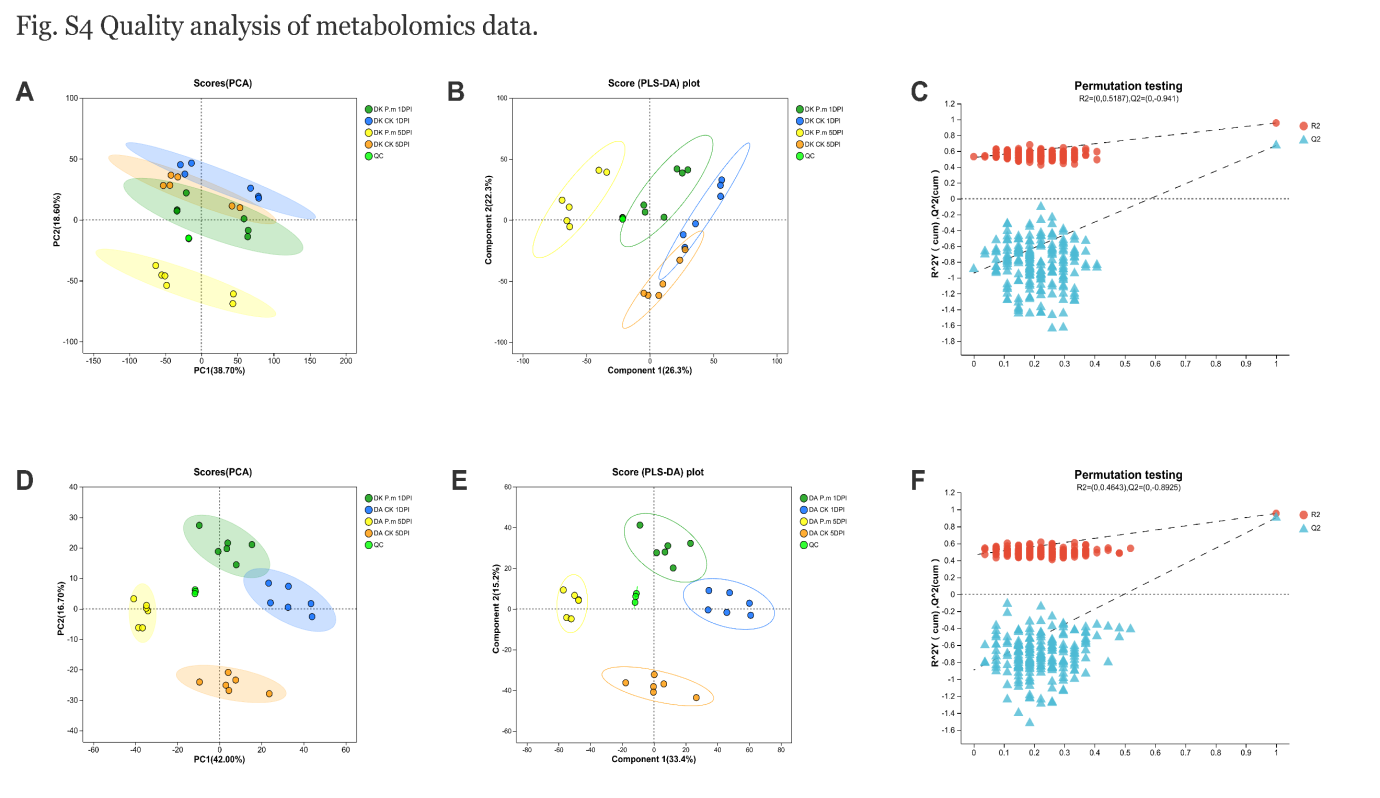


PCA score plot of samples acquired in Dongkui (A) and Dingao (D). PLS-DA score plot for Dongkui (B) and Dingao (E) samples, respectively. Confidence ellipses indicate that the groups of actual samples are distributed within the marked region with 95% confidence. PLS-DA permutation tests for Dongkui (C) and Dingao (F) samples: The x-axis represents the permutation number from the permutation test, while the y-axis displays the R² values (red dots) and Q² values (blue triangles). The dashed lines indicate the regression lines for R² and Q², respectively.
